# Progress in Neurology

**Published:** 1900-12-22

**Authors:** 


### PROGRESS IN NEUROLOGY.

**Myasthenia gravis.**—A valuable account of this rare affection has recently been published by Campbell and Bramwell.[4] The salient feature of the disease is weakness, sometimes amounting to complete paralysis of some or all of the voluntary muscles. After prolonged rest of the affected mucles they may respond normally to the will, but they become rapidly exhausted after voluntary contractions, regaining their power again after rest. In severe cases, however, weakness and even paralysis may be persistent even after prolonged rest. The affected muscles often exhibit the "myasthenic reaction," becoming exhausted by faradic stimulation, just as they are by voluntary contraction, galvanism, on the other hand, having little power in this respect. All the voluntary muscles may be affected, but those muscles are most apt to be implicated which act most constantly—*e.g.* the cervical and eye muscles. The bulbar muscles are very generally involved. A characteristic feature of the disease is its tendency to fluctuate in severity from day to day, and even to disappear for months. There are no sensory symptoms. Death occurs in a large proportion of the cases, but no structural changes have been found to account for the symptoms.

Males and females are almost equally affected. The age at which the disease begins varies. The youngest patient was 12 years, the oldest 55 years old. Syphilis and alcohol appear to play no part as causative factors. Not infrequently the symptoms have followed some previous affection of influenza.

The disease usually comes on gradually. Most frequently the characteristic weakness first shows itself in the muscles innervated by the bulb. Ptosis is a common symptom: it is usually bilateral, either constant or only present towards the latter part of the day. On account of the weakness of the occipito-frontales and of the cervical muscles, there is neither frontal overaction nor throwing back of the head to counteract this. Weakness of the external ocular muscles with consequent diplopia is often present. Pupil changes are quite exceptional. Difficulty in mastication is one of the most constant features of the disease; thi difficulty may be slight and only noticeable after a meal, and more especially towards evening. Sometimes the weakness of the muscles of mastication is very great, and the mouth is kept slightly open. The facial appearance is striking and characteristic, the face is immobile and expressionless, with drooping upper lids. The orbicularis palpebrarum is often weak. Difficulty in swallowing is rarely absent; and in several instances death has followed attacks of choking brought on whilst swallowing. Weakness of the palate is frequent, so that the voice becomes nasal and fluids may regurgitate through the nose. Paralysis of the laryngeal muscles has been but rarely noticed. A feeling of fatigue and aching in the tongue is often complained of. Characteristic alterations in speech occur. The patient speaks naturally when he begins, but soon speech becomes nasal, words become more and more indistinct and feeble, and finally he has to stop for want of breath. This is due partially to weakness of the articulatory apparatus and partially to defective action of the respiratory muscles. Weakness of the neck and trunk muscles is common. In the extremities the muscles situated nearest the trunk usually suffer the most. In slight cases the patient appears to walk quite naturally; when the legs feel tired he rests for a minute or two and then walks on almost as well as before.

The electrical reactions are perfectly characteristic; on applying a tetanising current to the muscles a brisk contraction is produced, but in a very short time the contraction gets less until finally the muscle ceases to respond. On reapplying the electrodes, after a little time, a good contraction is again obtained, which again dies away. Attempts to exhaust the muscles with galvanism results only in a very slight diminution of the contraction.

The muscular sense and co-ordination are never impaired. With exception of a feeling of fatigue in the muscles after they have been exercised, sensory symptoms are seldom present. The tendon jerks are usually brisk, and the plantar reflex is flexor. The sphincters are not effected, and no trophic changes occur. In the great majority of cases in which an autopsy has been made no lesion has been found, either in the central nervous system, in the nerves, or in the muscles.

We are therefore quite ignorant as to the true pathology of the disease. It may possibly be due to a poison, probably of microbic origin, acting on the lower motor neurons and interfering with their functional activity without producing discoverable change in structure. It is almost certainly some part, possibly the end plates, of the lower motor neuron, and not the upper neurons, that are affected, as shown by the myasthenic electrical reaction—the response to electrical stimulation dying away just as does that to voluntary effort.

The diagnosis in well-marked cases of myasthenia is easy, and can often be made at first glance from the facial expression, nasal speech, and ptosis. From bulbar paralysis the disease is distinguished by the presence of ptosis, upper facial weakness, paresis of the jaw and neck muscles and of the extremities, and by the myasthenic reaction. In hysteria and neurasthenia ready fatigue after exertion is met with, but the myasthenic reaction should enable a diagnosis to be arrived at. The variation in intensity of the symptoms is a diagnostic point of great importance.

The prognosis is, in any particular case, doubtful; nearly one half of those recorded have ended fatally; but some cases have been known to improve and remain free from symptoms for months and even years. And complete recovery has been known. Involvement of the respiratory muscles is of grave significance, and any pulmonary disorder is of serious import.

No specific treatment is known for the disease. Rest is of great importance, and excitement and cold are harmful. There is no evidence that drugs are of any service.

* Brain, Summer, 1900.

